# PCR-Based Detection of *Cryptosporidium* spp. and *Enterocytozoon bieneusi* in Farm-Raised and Free-Ranging Geese (*Anser anser f. domestica*) From Hainan Province of China: Natural Infection Rate and the Species or Genotype Distribution

**DOI:** 10.3389/fcimb.2019.00416

**Published:** 2019-12-04

**Authors:** Wei Zhao, Huan-huan Zhou, Tian-ming Ma, Jianping Cao, Gang Lu, Yu-juan Shen

**Affiliations:** ^1^National Institute of Parasitic Diseases, Chinese Center for Disease Control and Prevention, Shanghai, China; ^2^Chinese Center for Tropical Diseases Research, Shanghai, China; ^3^WHO Collaborating Centre for Tropical Diseases, Shanghai, China; ^4^National Center for International Research on Tropical Diseases, Ministry of Science and Technology, Shanghai, China; ^5^Key Laboratory of Parasite and Vector Biology, Ministry of Health, Shanghai, China; ^6^Department of Pathogenic Biology, Hainan Medical University, Haikou, China; ^7^Hainan Medical University-The University of Hong Kong Joint Laboratory of Tropical Infectious Diseases, Hainan Medical University, Haikou, China; ^8^Key Laboratory of Tropical Translational Medicine of Ministry of Education, Hainan Medical University, Haikou, China; ^9^Department of Parasitology, Wenzhou Medical University, Wenzhou, China

**Keywords:** *Cryptosporidium*, *Enterocytozoon bieneusi*, PCR-based detection, goose, Hainan **(China)**

## Abstract

*Cryptosporidium* spp. and *Enterocytozoon bieneusi* are two important zoonotic pathogens that can infect humans and a broad range of animal hosts. However, few studies have been conducted to study infection of the two pathogens in domestic geese until now. The aims of the present study were to determine the prevalence of natural infection, and the species or genotype distribution of *Cryptosporidium* and *E. bieneusi* in farm-raised and free-ranging geese from Hainan Province of China. In total, 266 fecal samples of geese were collected (142 farm-raised and 124 free-ranging geese). *Cryptosporidium* spp. and *E. bieneusi* were identified by nested PCR and sequencing analysis of the SSU rRNA and the ITS region of the rRNA genes. A total of 4.1% (12/226) of the geese were positive for *Cryptosporidium* spp., with 0.7% identified in the farm-raised geese and 7.0% in the free-ranging geese. Two bird-adapted species/genotypes were identified: *C. baileyi* (*n* = 1) and *Cryptosporidium* goose genotype I (*n* = 11). Meanwhile, *E. bieneusi* was found in 13.9% (37/266) of geese, with 8.9% identified in the farm-raised and 21.8% in the free-ranging geese. Eleven genotypes of *E. bieneusi* were identified constituted with six known genotypes: D (*n* = 13), I (*n* = 5), CHG2 (*n* = 1), CHG3 (*n* = 5), and CHG5 (*n* = 1), and five novel genotypes named HNE-I to V (one each). All of the genotypes identified in the geese here belonged to zoonotic Groups 1 or 2. This study is the first to demonstrate the presence of *Cryptosporidium* spp. and *E. bieneusi* in domestic geese from Hainan, China, and provides baseline data that will be useful for controlling and preventing these pathogens in goose farms. The geese infected with *E. bieneusi*, but not with *Cryptosporidium*, should be considered potential public health threats.

## Introduction

*Cryptosporidium* spp. and *Enterocytozoon bieneusi* are two obligate intracellular pathogens, which are common etiological agents of diarrhea in humans and animals around the globe (Fayer and Santin-Duran, 2014; Checkley et al., 2015). Both pathogens can cause death diarrhea in immunocompromised people, and are responsible for significant morbidity and mortality of children in developing countries (Matos et al., 2012; Khalil et al., 2018). *Cryptosporidium* and *E. bieneusi* can infect numerous vertebrate animal hosts, including mammals, birds, amphibians, reptiles, and fish (Santín and Fayer, 2011; Pumipuntu and Piratae, 2018). More importantly, the infective oocysts or spores of these two pathogens are ubiquitous in the environment. They are transmitted in humans, potentially through the fecal-oral route, either directly, via contact with infected humans or animals, or indirectly by ingesting food or water contaminated with the pathogens (Fayer and Santin-Duran, 2014; Xiao and Feng, 2017). Despite this knowledge, the contribution of each animal source to human infections is poorly understood.

Research conducted on the molecular epidemiology of *Cryptosporidium* has shown that it has a high diversity at the species level (Feng et al., 2018). To date, a total of 39 species, and more than 70 genotypes of *Cryptosporidium* have been described and 21 species and four genotypes out of these have been reported in humans (Feng and Xiao, 2017; Holubová et al., 2019). Many human-pathogenic species or genotypes have also been found in a variety of animal species, including farm animals, pets, and wildlife (Pumipuntu and Piratae, 2018). Similarly, *E. bieneusi* is a complex species of Microsporidia, and more than 500 ITS genotypes have been reported in humans and various animal (Li et al., 2019a). These genotypes can be placed into 11 distinct groups (named Groups 1–11) by phylogenetic analysis (Li et al., 2019b). More than 90% of the genotypes belong to Groups 1 or 2. Some of these genotypes are probably responsible for most zoonotic infections, and constitute a major risk for zoonotic or cross-species transmission because they have a large variety of hosts, including humans. The other nine groups (Groups 3–11) mainly include genotypes from specific hosts or wastewater, showing host adaption to some extent (Guo et al., 2014; Li et al., 2019b). The sources of contamination of *Cryptosporidium* spp. and *E. bieneusi* infection in humans can be clarified by genotyping the two pathogens in different hosts.

The majority of domesticated geese (*Anser anser domesticus*) descend from the Greylag goose (*Anser anser*). Geese are bred for meat, eggs, and feathers, and 95.3% of goose production in 2017 was in Asia (http://www.fao.org/faostat/en/#data/QL). The farming of geese has a long history in China. However, there are no available reports on identification and genotyping of *Cryptosporidium* in geese in China. Currently, only one study has evaluated *E. bieneusi* in geese, but the study included a small sample size and was limited to a very narrow geographical area of China (Zhao et al., 2016). The role of geese in the transmission of *Cryptosporidium* spp., and *E. bieneusi* remains unclear. The aims of the present study were to determine the prevalence of natural infection of *Cryptosporidium* and *E. bieneusi* in farm-raised and free-ranging geese from Hainan Province of China, to identify the genotype of *Cryptosporidium* and *E. bieneusi* isolates, and to assess the potential zoonotic transmission by homology and phylogenetic analysis.

## Materials and Methods

### Ethics Statement

Before beginning work on the present study, we contacted the farm owners and obtained their permission to have their animals involved. Written informed consent was obtained from the owners for the participation of their animals in this study. The protocol was also reviewed and approved by the Ethics Committee of Hainan Medical University.

### Collection of Fecal Specimens

**Figure 1:**
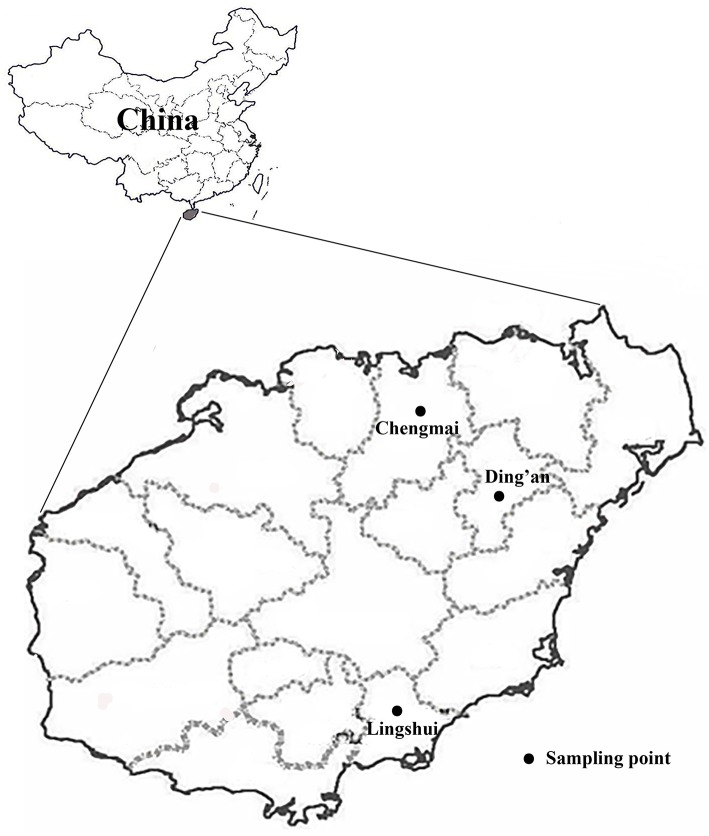
Specific locations where samples were collected in this study.

During the period from March to July of 2019, a total of 266 fresh fecal specimens (~10 g of each) were collected from 142 farm-raised and 124 free-ranging geese from three farm: Chengmai (*n* = 67), Ding'an (*n* = 45), and Lingshui (*n* = 30) cities and two areas (Chengmai city) in Hainan Province of China (Figure 1 and Table 1). The farms of geese were selected based only on the owners' willingness to participate and the accessibility of animals for sampling. All of the fecal specimens were collected from the ground immediately after defecation using a sterile disposable latex glove. Each individual sample was then placed in a labeled, sterile bag. To avoid duplicate sampling, only one fecal specimen was collected from each animal. None of the animals had apparent clinical signs at the time of sampling. Samples were obtained from 10% of the total number of geese in each farm or area. All of the specimens were transported to the laboratory in a cooler with ice packs (<24 h) and stored at −20°C until processing (<1 w).

**Table 1:** Prevalence and *Cryptosporidium* species/genotypes and *E. bieneusi* genotypes in goose according to feeding mode.

**Groups**	**No. of examined**	***E. bieneusi***		***Cryptosporidium*** **spp**.	
		**Prevalence**	**Genotypes (*n*)**	**Prevalence**	**Species/genotype (*n*)**
**Farm-Raised**					
Farm1 (Chengmai)	67	7 (9.0)	CHG5 (3); HNE-II to V (each one)	0	–
Farm2 (Ding'an)	45	0	–	1 (2.2)	Goose genotype I (1)
Farm3 (Lingshui)	30	3 (10.0)	CHG3 (3)	0	–
Subtotal	142	10 (7.0)	CHG3 (3); CHG5 (3); HNE-II to V (each one)	1 (0.7)	Goose genotype I (1)
**Free-Ranging**					
Area1 (Chengmai)	56	12 (21.4)	D (8); CHG2 (1); CHG3 (1); CHG5 (1); HNE-I (1)	6 (10.7)	*C. baileyi* (1); Goose genotype I (5)
Area2 (Chengmai)	68	15 (22.1)	I (5); D (5); BEB6 (3); CHG3 (1); CHG5 (1)	5 (7.4)	Goose genotype I (5)
Subtotal	124	27 (21.8)	D (13); I (5); BEB6 (3); CHG3 (2); CHG5 (2); CHG2 (1); HNE-I (1)	11 (8.9)	*C. baileyi* (1); Goose genotype I (10)
Total	266	37 (13.9)	D (13); I (5); BEB6 (3); CHG3 (5); CHG5 (5); CHG2 (1); HNE-I to V (each one)	12 (4.1)	*C. baileyi* (1); Goose genotype I (11)

### DNA Extraction

Each of the feces samples was thoroughly mixed with 40 mL of distilled water, and the liquid was then concentrated by centrifugation at 1,500 g for 10 min. Genomic DNA was directly extracted from ~200 mg of each processed fecal specimen using a QIAamp DNA stool mini kit (QIAgen, Hilden, Germany), according to the manufacturer-recommended procedures. Then, 200 μL of extracted DNA from each sample was transferred into Eppendorf tubes, and stored at −20°C until PCR amplification.

### PCR Amplification

*Cryptosporidium* in the fecal specimens was identified by nested PCR amplification of a SSU rRNA gene fragment of ~830 bp. The primers and the cycle parameters were designed by Xiao et al. (1999). Meanwhile, all DNA preparations were analyzed for the presence of *E. bieneusi* by amplifying an ~390 bp region of the rRNA gene of *E. bieneusi*. The primers and the cycle parameters were designed by Buckholt et al. (2002). TaKaRa Taq DNA Polymerase (TaKaRa Bio Inc., Tokyo, Japan) was used for all PCR amplifications. PCR amplifications were performed with positive controls (rat-derived *Cryptosporidium* rat genotype IV DNA for *Cryptosporidium* spp. and rat-derived genotype Peru 8 DNA for *E. bieneusi*) and negative controls (2 μL distilled water). All of the secondary PCR products were subjected to electrophoresis in a 1.5% agarose gel, and were visualized by staining the gel with GelRed (Biotium Inc., Hayward, CA).

### Nucleotide Sequencing and Analyzing

All of the *Cryptosporidium* and *E. bieneusi* positive PCR products were sent to Sangon Biotech Co. Ltd. (Shanghai, China), for sequencing, which all of the samples were sequenced in both directions. The species or genotypes of *Cryptosporidium* and the genotypes of *E. bieneusi* were identified by comparing the nucleotide sequences obtained with each other and with published GenBank sequences using the Basic Local Alignment Search Tool (BLAST) (https://blast.ncbi.nlm.nih.gov/Blast.cgi) and ClustalX 1.83 (http://www.clustal.org/).

### Phylogenetic Analysis

To confirm the genogroup designation and to assess the genetic relationships of novel ITS genotypes of *E. bieneusi* obtained, Bayesian inference (BI) and the Monte Carlo Markov Chain (MCMC) method were used to construct phylogenetic trees in MrBayes v 3.2.6 (http://mrbayes.sourceforge.net/). Fig Tree v 1.4.4 (http://tree.bio.ed.ac.uk/software/fifigtree/) was used to visualize and edit the maximum clade credibility tree generated by these analyses. Posterior probability values were estimated based on 1,000,000 generations with four simultaneous tree building chains, with trees being saved every 100th generation. A 50% majority rule consensus tree for each analysis was constructed based on the final 75% of trees generated by BI.

### Statistical Analysis

Data entry and analysis were performed using Social Sciences (SPSS) 19.0 software. The statistical significance of differences in infection proportions was generally evaluated by Pearson's Chi-square test. The significant level of all tests was: *p* = 0.05.

### Nucleotide Sequence Accession Numbers

Representative nucleotide sequences obtained in the study were deposited in the GenBank database under accession numbers MN472907 to MN472911 for *E. bieneusi*; MN461548 and MN461549 for *Cryptosporidium*.

## Results

### Occurrence of *Cryptosporidium* and *E. bieneusi* in Geese

The prevalence of *Cryptosporidium* and *E. bieneusi* in geese were 4.1% (12/226) and 13.9% (37/266), respectively. In farm-raised geese, one out of three farms were *Cryptosporidium*-positive and two out of three farms were *E. bieneusi*-positive. Both of the two areas of free-ranging geese were positive for both *Cryptosporidium* and *E. bieneusi*. The prevalence of *Cryptosporidium* and *E. bieneusi* in farm-raised geese were lower than in free-ranging geese (0.7% vs. 7.0%, χ^2^ = 7.71, *P* = 0.01; and 8.9% vs. 21.8%, χ^2^ = 7.96, *P* = 0.008) (Table 1).

### *Cryptosporidium* Species/Genotypes Identified in Geese

All of the 12 *Cryptosporidium-*positive specimens were successfully sequenced at the SSU rRNA locus. Using sequence analysis, *C. baileyi* (*n* = 1) and *Cryptosporidium* goose genotype I (*n* = 11) were identified. The *C. baileyi* sequence obtained had 100% homology with a sequence (JX548296) obtained in chickens in Zhejiang, China. All of the 11 sequences of *Cryptosporidium* goose genotype I were identical, and had not previously been reported, although they had only one base different from the sequence (AY120912) found in an isolate of Canada geese from the USA. In the present study, *Cryptosporidium* goose genotype I showed predominance and it was found in one farm-raised goose and 10 free-ranging geese. While, *C. baileyi* was only found in a free-ranging goose (Table 1).

### Genetic Characterizations and Genotypic Distribution of *E. bieneusi* in Geese

**Figure 2:**
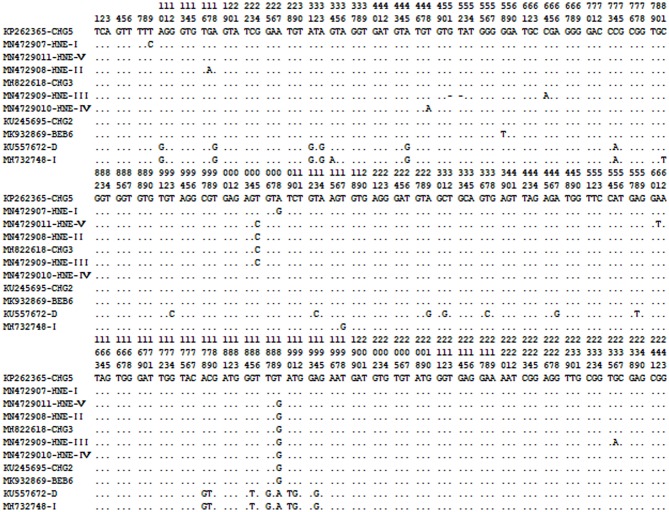
Sequence variation in the ITS region of the rRNA gene of *E. bieneusi* isolates identified in geese here. Dots indicate the same base identity as the ITS gene sequence of genotype CHG5 (KP262365).

Sequencing analysis of the 37 *E. bieneusi* isolates in this study showed that 11 different representative sequences were identified, with a total of 33 polymorphic sites observed (Figure 2). Six of 11 representative sequences had been previously reported, and were identical to the genotypes D, I, BEB6, CHG3, CHG5, and CHG2. However, the other five ITS gene sequences (MN472907 to MN472911) had not been previously reported, and were named genotypes HNE-I to HNE-V. HNE-I and HNE-IV had 99.18 and 99.59% homology with the genotypes CHG5 (KP262365) and CHG2 (KT235708), respectively, while genotypes HNE-II, HNE-III and HNE-V had 99.59, 98.35, and 99.59% homology with genotype CHG3 (MH822618), respectively.

Out of the 11 genotypes, genotype D was the most prevalent, with a prevalence of 57.3% (13/37), followed by genotype I, CHG3 and CHG5, which each had a prevalence of 28.0% (5/37), BEB6 with a prevalence of 4.9% (3/37), and then the remaining six genotypes, CHG2 and HNE-I to V, which each had a prevalence of 1.2% (1/37) (Table 1). Genotypes D, I, BEB6, CHG2, and HNE-I were only found in free-ranging geese, and genotypes HNE-II to V were only found in farm-raised geese, while genotypes CHG3 and CHG5 were found in both farm-raised and free-ranging geese. The distributions of *E. bieneusi* genotypes in animals characterized by feeding mode was shown in Table 1.

### Phylogenetic Relationship of *E. bieneusi* Genotypes

**Figure 3:**
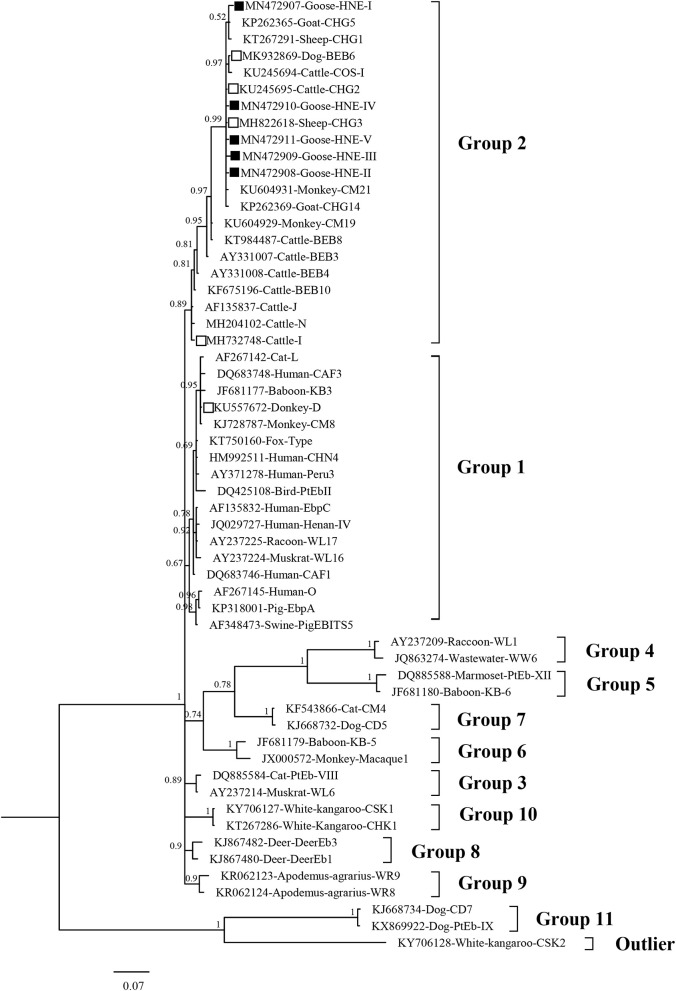
Bayesian phylogenetic analysis of *Enterocytozoon bieneusi* ITS sequences. Phylogenetic relationship of *E. bieneusi* genotypes identified here and other known genotypes deposited in GenBank was inferred by a Bayesian phylogenetic analysis of ITS sequences based on genetic distance by the Bayesian inference (BI) and the Monte Carlo Markov Chain (MCMC) method. The statistically significant posterior probabilities are indicated at branches. Each sequence is identified by its accession number, host origin, and genotype designation. The *E. bieneusi* genotype CSK2 (KY706128) from white kangaroo was used as the outgroup. The empty squares and the squares filled in black indicate known and novel genotypes identified in this study, respectively.

In the phylogenetic analysis tree, genotype D was classified in Group 1, and the genotypes I, BEB6, CHG2, CHG3, CHG5, and HNE-I to V were in Group 2 (Figure 3).

## Discussion

The first report on goose cryptosporidiosis was published in 1974, and identified parasites in five 14-day-old goslings from Iowa, USA (Proctor and Kemp, 1974). Since then, only two reports on *Cryptosporidium* infections in domestic geese have been published. In one study, *Cryptosporidium* infection was detected in 44 (59%) of goslings aged 8–35 days (Richter et al., 1994). In the other study, *Cryptosporidium* infection was investigated in geese experimentally infected with Usutu virus using *situ*-hybridization, and showed that 89% of conjunctival tissue samples and 88% of bursal tissue samples of these geese were *Cryptosporidium*-positive (Chvala et al., 2006). Furthermore, *Cryptosporidium* was also reported in Canada geese in two studies conducted in the USA, with infection rates of 6.8 and 23.4% (Jellison et al., 2004; Zhou et al., 2004). For *E. bieneusi*, only one study has been reported in geese from Heilongjiang, China with a 30.8% (8/26) infection rate (Zhao et al., 2016). In the present study, the prevalence of *Cryptosporidium* spp. and *E. bieneusi* in geese were 4.1% (12/226) and 13.9% (37/266), respectively. The infection rates of both *Cryptosporidium* and *E. bieneusi* were higher in free-ranging geese than in farm-raised geese, with 7.0% for *Cryptosporidium* and 21.8% for *E. bieneusi* in free-ranging geese, and 0.7% for *Cryptosporidium* and 8.9% for *E. bieneui* in farm-raised geese. The higher infection rates of *Cryptosporidium* and *E. bieneusi* in free-ranging geese could be explained by the fact that farmed geese have better hygiene, whereas free-ranging geese have a wide range of space for activity, and access to a variety of water sources.

There have been a few studies on the identity of *Cryptosporidium* spp. in geese, which have identified the occurrence of three species, including human pathogens *C. hominis* and *C. parvum*, and the avian species *C. baileyi* (Graczyk et al., 1998; Zhou et al., 2004; Chvala et al., 2006; Jellison et al., 2009). Meanwhile, six *Cryptosporidium* genotypes have also been found in geese, including four goose-specific genotypes named goose genotype I to IV, and the zoonotic pathogen muskrat genotype I, as well as *Cryptosporidium* duck genotype and *C. hominis*-like genotype (Zhou et al., 2004). However, only one study has characterized *Cryptosporidium* spp. in domestic geese, and only *C. baileyi* was found in those animals (Chvala et al., 2006). While, non-genotyped cryptosporidia have been detected in the swan goose and the black swan (Rohela et al., 2005). In the present study, *C. baileyi* and *Cryptosporidium* goose genotype I was identified in the domestic geese. *C. baileyi* was *first* identified in chickens (Current et al., 1986). Evidence has shown that *C. baileyi* can infect a broad range of birds with a wide geographic distribution (Nakamura and Meireles, 2015). Although *C. baileyi* has been identified in one immunodeficient patient, this species should not be considered as a true zoonotic agent since this patient was immunodeficient, and no other reports exist (Ditrich et al., 1991). *Cryptosporidium* goose genotype I has been commonly found in Canada geese, and has also been found in aquatic birds and peafowl (Jellison et al., 2004; Zhou et al., 2004; Cano et al., 2016; Feng et al., 2019). To date, no human cases of cryptosporiosis caused by *Cryptosporidium* goose genotype I have been identified. As only a small number of isolates of *Cryptosporidium* goose genotype I were isolated, the host specificity of the genotype is not yet fully understood. The source of this genotype infection and its transmission dynamics now require further investigation to elucidate the cross-species transmission potential of *Cryptosporidium* goose genotype I in geese and other birds in China.

One study revealed the presence of three genotypes (BEB6, Peru 6, and CHN-B3) of *E. bieneusi* in geese from Heilongjiang, China (Zhao et al., 2016). BEB6 and Peru 6 are known human-pathogenic genotypes, and genotype CHN-B3 belongs to Group 1, and therefore has a potential for zoonotic transmission (Zhao et al., 2016). In the present study, 11 *E. bieneui* genotypes were identified, including six known genotypes (D, I, BEB6, CHG3, CHG5, and CHG2) and five novel genotypes (HNE-I to HNE-V). Of these, genotype D, I, and BEB6 have been found in humans, and genotypes CHG2, CHG3, and CHG5 were commonly found in goats and sheep in China (Shi et al., 2016; Chen et al., 2018; Yang et al., 2018; Li et al., 2019a). Out of the 11 genotypes identified in the present study, genotype D is the most frequently detected in humans, and has been found in both HIV-positive patients and HIV-negative individuals from America, Europe, Asia, and Africa (Matos et al., 2012). Genotype D appears to have a wide host range as it has also been isolated from a large variety of animals including cattle, cats, horses, dogs, and some wild animals (macaques, muskrats, raccoons, beavers, foxes, and gorillas) as well as in birds, such as pigeons and falcons (Zhao et al., 2014; Li et al., 2019a). This genotype was the most common genotype found in geese in the present study, with a prevalence of 57.3% (13/37). The results above suggest that domestic geese infected with genotype D may transmit it to other animals and humans. Genotypes I and BEB6 are commonly found in cattle as well as in children from Shanghai and Changchun of China (Zhang et al., 2011; Wang et al., 2013; Zhao et al., 2015). Thus, the geese infected with those genotypes also have a potential to cause environmental pollution and human infection. To date, Genotypes CHG2 and CHG3 have been found in goats, sheep, and cattle (Li et al., 2016; Shi et al., 2016; Yang et al., 2018; Li W. C. et al., 2019; Udonsom et al., 2019), and genotype CHG5 was only found in goat (Shi et al., 2016). This was the first report of genotypes CHG2, CHG3, and CHG5 in geese indicating that those genotypes have an extensive host range. The five novel genotypes were included in Group 2. To date, the potential of genotypes CHG2, CHG3, and CHG5 and the five novel genotypes (HNG-I and HNG-II) to cause disease in humans or other livestock is unknown. Their host adaptation and potential role in the zoonotic transmission of *E. bieneusi* infection now requires further exploration in more systematic molecular epidemiological investigations of *E. bieneusi* in a larger number of hosts.

## Conclusion

The present study is the first to demonstrate the occurrence and molecular characterizations of *Cryptosporidium* and *E. bieneusi* in farm-raised and free-ranging geese from the Hainan Province of China. Two bird-adapted species (*C. baileyi*) or genotypes (*Cryptosporidium* goose genotype I) of *Cryptosporidium* were identified. Thus, there is a lower risk of zoonotic transmission of *Cryptosporidium* between geese and humans in the areas investigated. However, all of the 11 genotypes of *E. bieneusi* were belonged to Groups 1 or 2, which are considered potentially zoonotic. Additionally, the three zoonotic *E. bieneusi* genotypes (D, I, and BEB6) were found in 56.8% of the geese that were tested. These results suggest that the geese infected with these genotypes of *E. bieneusi* have the risk of zoonotic potential and pose a threat to human health.

## Data Availability Statement

The datasets generated for this study can be found in the Representative nucleotide sequences obtained in the study were deposited in the GenBank database under accession numbers MN472907 to MN472911 for *E. bieneusi*; MN461548 and MN461549 for *Cryptosporidium*.

## Author Contributions

YS and GL conceived and designed the experiments. WZ, HZ, and TM performed the experiments. WZ analyzed the data and wrote the paper. JC contributed the reagents materials analysis tools. YS and GL critically revised the manuscript. All authors read and approved the final version of the manuscript.

### Conflict of Interest

The authors declare that the research was conducted in the absence of any commercial or financial relationships that could be construed as a potential conflict of interest.
